# Genetic variants of the *EGFR* ligand-binding domain and their association with structural alterations in Arab cancer patients

**DOI:** 10.1186/s13104-021-05559-y

**Published:** 2021-04-19

**Authors:** Maryam Marzouq, Ali Nairouz, Noureddine Ben Khalaf, Sonia Bourguiba-Hachemi, Raed Quaddorah, Dana Ashoor, M. Dahmani Fathallah

**Affiliations:** 1 Department of Life Sciences, Health Biotechnology Program, College of Graduate Studies, Arabian Gulf University, PO Box 26671 Manama, Kingdom of Bahrain; 2 King Fahad Chair for Health Biotechnology, College of Graduate Studies, Arabian Gulf University, PO Box 26671 Manama, Kingdom of Bahrain

**Keywords:** EGFR protein, Missense mutation, Arabs, Adenocarcinoma, Single nucleotide polymorphism, CR1/CR2 EGFR domains, EGFR R521K polymorphism, Arabian Peninsula population

## Abstract

**Objective:**

This study aimed to identify novel genetic variants in the CR2 extracellular domain of the epidermal growth factor receptor (EGFR) in healthy individuals and patients with six different types of adenocarcinoma, in Arabian peninsula populations. It also aimed to investigate the effects of these variants on the EGFR structure and their eventual relevance to tumorigenesis.

**Results:**

We detected seven new EGFR genetic variants in 168 cancer patients and 114 controls. A SNP rs374670788 was more frequent in bladder cancer but not significantly associated to. However, a missense mutation (V550M) was significantly associated to colon, ovary, lung, bladder and thyroid cancer samples (p < 0.05). Three mutations (H590R, E602K and T605T) were found in the heterozygous form only in colon cancer patients. Genomic analysis of the synonymous mutation (G632G) showed that the T/A genotype could be associated to thyroid cancer in Arab patients (p < 0.05). An additional novel SNP rs571064657 was observed in control individuals. Computational analysis of the genetic variants revealed a reduction in the stabilization of the EGFR tethered form for both V550M and the common R521K variant with low energetic state (− ∆∆G). Molecular interactions analysis suggested that these mutations might affect the receptor’s function and promote tumorigenesis.

**Supplementary Information:**

The online version contains supplementary material available at 10.1186/s13104-021-05559-y.

## Introduction

It has been reported that cancer prevalence is increasing in the Arabian peninsula populations [1]. This trend was linked to a number of contributing factors such as increased life expectancy, diet, young smoking, obesity, and pollution [2, 3]. These observations spurred better cancer patients characterization particularly at the level of cancer markers such as the epidermal growth factor receptor (EGFR). Indeed, upon binding to ligands like EGF or TGF-α, the EGFR undergoes autophospholrylation that leads to the activation of several signal-transduction cascades, and cell cycle-progression [4]. Furthermore, molecular alterations of the EGFR activate pro-oncogenic signaling pathways, including the RAS-RAF-MEK-ERK MAPK and AKT-PI3K-mTOR [5]. In these cases, the activation of EGFR turns on tumorigenesis promoting event such as cellular proliferation, resistance to apoptosis, and angiogenesis [6, 7]. Alongside, the *EGFR* gene amplification leads to the receptor overexpression, accelerating its uncontrolled activation which is associated with several malignancies including lung, breast, stomach, colorectal, head and neck, pancreatic carcinomas and glioblastoma [8–11]. The *EGFR* gene encodes a protein that have different functional domains with exons 1 to 16 encoding the extracellular ligand-binding domain [8]. The later contains 4 sub-domains: 1 = L1, II = CR1, III = L2, and IV = CR2. L1 and L2 are leucine-rich ligand-binding domains. Whereas, CR1 and CR2 are cysteine-rich and involves the formation of structurally important disulfide bonds [7–9]. EGFR has a tethered monomeric form and an extended dimeric complex. X-ray crystals and molecular dynamic simulations studies described these structural changes and linked them to EGFR regulation in cancer [12–14]. In the tethered conformation, EGFR ectodomain II and IV are folded into each other’s forming the hairpin loop of domain II spanning residues 240–260 and interacts with C1IVc and C1IVd modules of domain IV (spanning residues 561–569 and 572–585, respectively). EGF receptor is concomitant to domains rearrangement in a way that domain I and III are accessible for EGF and when domain II dimerizes with another EGFR unfolded tethered form that rotated 90° on its vertical axis. Alterations of the extracellular domains’ protein sequences have been related to cancer prevalence and the effectiveness of targeted immunotherapy [14, 15]. Indeed, variant R521K is a widespread functional variant that plays an important role in EGFR expressing tumors and impacts the effectiveness of anticancer agents [16, 17].

## Main text

### Methods

#### Study population

168 Cancer patients from the Arabian peninsula populations (N = 74 colon, 20 breast, 20 ovary, 20 bladder, 14 lung, 20 thyroid) alongside 114 healthy controls matched for gender, age, and ethnicity.

#### Genomic DNA isolation

DNA was extracted from FFPE tissues (patients) and peripheral blood (controls) using QIAamp DNA FFPE Tissue Kit (Cat#0.56404) and QIAamp DNA Mini Kit (Cat#0.51306) respectively, according to the manufacturer’s instructions. DNA samples were stored at – 20 °C.

#### Mutational analysis

PCR was used to amplify the exons coding for EGFR-CR2 domain using primers [18] and conditions as shown in Additional file 1. PCR was conducted in 50 µl containing 33 ng of DNA, 5 × GoTaq buffer, 0.2 mM dNTP mixture, 4 mM MgCl_2_, 0.025 µlM each primer, 0.96 U/µl GoTaq Enzyme, and nuclease-free water. PCR products were checked on 2% agarose gel and sequenced using the dye termination method [19]. All detected gene alterations were confirmed by two independent sense and antisense PCR. Some samples were dropped because one of the two PCR reactions failed to confirm the nucleotide change. Indeed, the extraction of high-quality DNA (suitable for PCR reactions and sequencing) from some cancer samples was certainly affected by the storage of FFPE biopsy specimens (old blocks).

#### Bioinformatics study

##### Model preparation

The untethered structure of EGFR (id:3NJP) and the tethered EGFR/EGF complex structure (id:1NQL) were downloaded from the RCSB PDB database (https://www.rcsb.org/). The Pymol program (Schrödinger) was used for structure visualization and mutants models generation.

##### Interaction analysis for energy minimization

Wild type and mutants (R521K,V550M) forms of tethered EGFR/EGF complexes were solvated in a periodic boundary cube of water (2.4 Å × 2.4 Å × 2.4 Å) using VMD solvation plugin [20]. NaCl ions were added to neutralize the molecule. The steepest descent energy minimization was applied to relax the structures at 310 K (50,000 steps) in NVT mode, using NAMD (Nanoscale Molecular Dynamics program; v 2.9) [21]. Average structures obtained with UCSF Chimera (UCSF, USA) were analyzed for inter-chain residue H-bond interactions with LigPlot + [22].

##### Protein stability prediction

Site directed mutator (SDM) server (http://marid.bioc.cam.ac.uk/sdm2) was used to predict the effect of the mutations on protein stability. The variation of amino acid replacements that occurs a specific structural environment is analyzed by SDM according to substitution probability tables, generated from tolerated substitution in homologous proteins with known 3D-structures [23].

### Statistical analysis

Standard contingency table and Chi-square test were used to assess the association of the genotype and allele frequencies of the EGFR-CR2 variants with cancer. A *p*-value < 0.05 was considered statistically significant.

## Results

### Gene alterations analysis

**Fig. 1:**
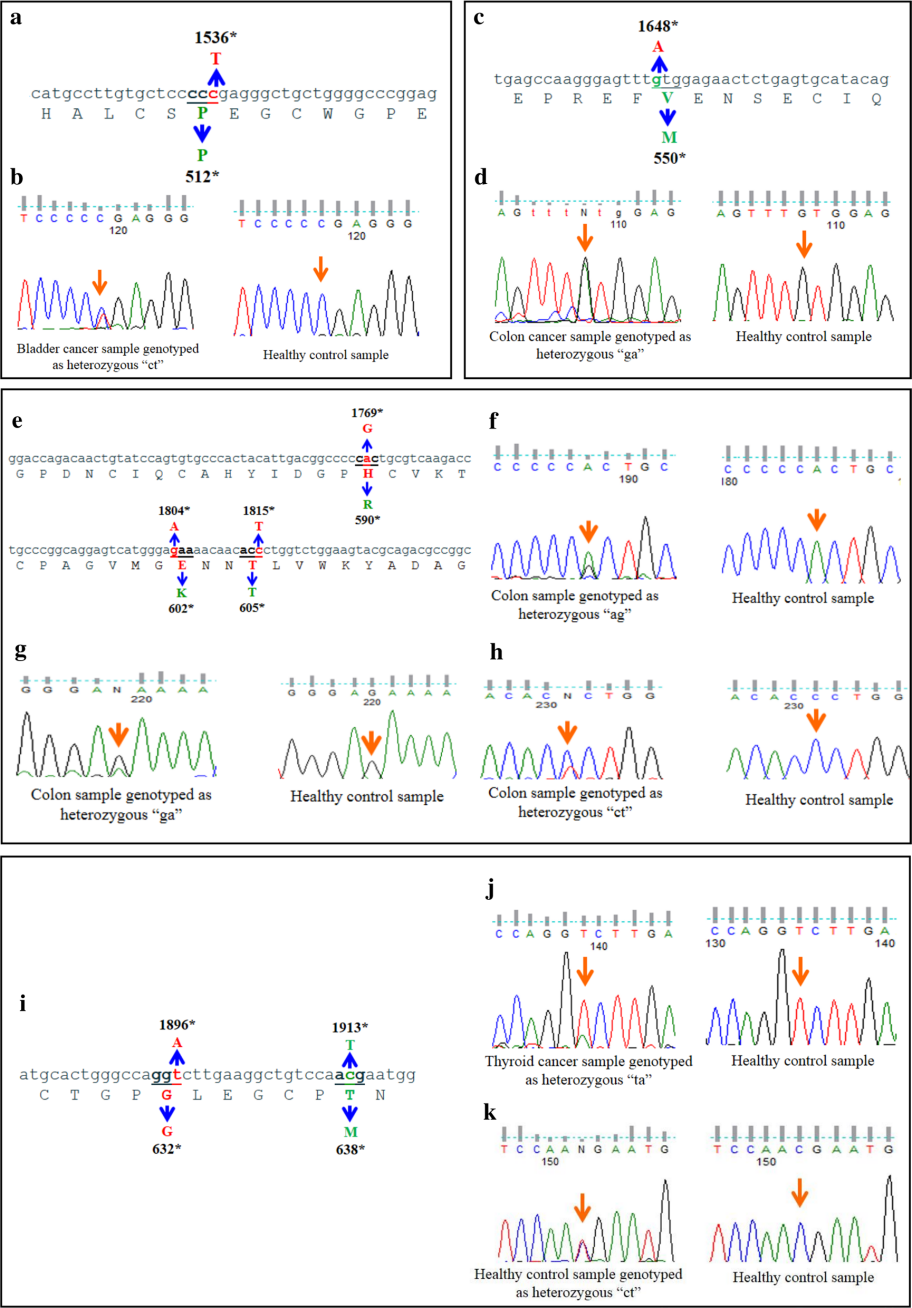
**The new variants in CR-2 domain of *EGFR* gene in cancer patients and healthy subjects**. **a** Alignment of nucleic and amino acid sequences of exon 13 of the *EGFR* gene showing the new SNP 1536C > T (P512P) marked with an asterisk. **b** Chromatographic patterns of direct sequencing showing new mutation in exon 13 of EGFR, 1536 C > T (P512P) (indicated by the arrows). **c** Alignment of nucleic and amino acid sequences of exon 14 of the *EGFR* gene showing the novel mutation 1468G > A (V550M) marked with an asterisk. **d** Chromatographic patterns of direct sequencing of EGFR exon 14 showing the novel mutation 1648 G > A (V550M) (pointed by an arrows). **e** Alignment of nucleic and amino acid sequences of exon 15 of the *EGFR* gene showing the new mutations 1769A > G (H590R), 1804G > A (E602K), and 1815C > T (T605T) respectively, all marked with an asterisk. **f–h** Chromatographic patterns of direct sequencing of EGFR exon 15 showing the novel mutations 1769**A** > **G** (H590R), 1804**G** > **A** (E602K), and 1815**C** > **T** (T605T) resepectively, the sequence change detected pointed by an arrows. **i** Alignment of nucleic and amino acid sequences of exon 16 of the *EGFR* gene showing two new variants 1896T > A (G632G) and 1913C > T (**T**638**M**) respectively marked with an asterisk. **j**, **k** Chromatographic patterns of direct sequencing of EGFR exon 16 showing the new mutation 1896 T > A (G632G) and the novel SNP1913 C > T (**T**638**M**), respectively

In ***exon 13***, we found a novel SNP, a transition 1536**C > T** yielding synonymous substitution (P512P) (Table 1; Fig. 1a). This new SNP was assigned the ID rs374670788 by the dbSNP (ss825678873) data bank. Although the frequency of the heterozygous variant CT (Table 1; Fig. 1b) was high in bladder sample (6% vs. 1% in control), no statistically significant association was observed (*p* = 0.231). Genotypes distributions of tested polymorphisms were consistent with the Hardy–Weinberg equilibrium (p > 0.05). Also, the presence of 5 reported variants were confirmed (Additional file 2), including the common variant rs2227983, **R**521**K** which has been reported in NCBI-ClinVar database as related to cancer [24, 25]. There was no association of SNP rs2227983 with any cancer type, 47.9% in patients versus 47.4% in controls (Additional file 2). In ***exon 14***, we observed a novel missense mutation (1648**G > A**) resulting in the substitution (V550M) (Table 2; Fig. 1c, d). The rate of heterozygous **GA** variant was statistically significant in colon, lung, ovary, bladder, and thyroid tumor samples (*p* < 0.05). Also, one reported SNP was observed (Additional file 2). In ***exon 15,*** three new mutations were detected (1769**A > G**, 1804**G > A**, and 1815**C > T**) in cancer patients but not in healthy subjects (Table 1; Fig. 1e) and confirmed by reverse sequencing. These alterations yielded respectively the missense mutations H590R, E602K and T605T synonymous substitution (Fig. 1f–h, respectively). Also, three reported SNPs were identified (Additional file 2). The frequency of the heterozygous variant rs17290169, was statistically highly significant in controls (*p* < 0.05). In ***exon 16***, two novel alterations were found: a 1896**T > A**, transversion (Table 1; Fig. 1i) that was detected in 3.1% of colon tumor samples, 10% of ovary tumor samples and 44.4% of thyroid tumor samples (Table 1; Fig. 1j). The frequency of heterozygous (**T/A)** variant was statistically highly significant in thyroid tumor samples (*p* < 0.05). Likewise, we observed a new SNP 1913**C > T**, a transition yielding synonymous substitution **T**638**M** (Fig. 1i). We observed this rare allele only in 2 controls (Fig. 1k). The newly identified SNP was assigned the ID rs571064657, by the dbSNP ss825678874 data base. In addition, one reported SNP was detected (Additional file 2).

**Table 1:** Novel genetic alterations in CR2 domain: allele and genotype frequencies

Exon	SNP ID	Type of Samples (n)	Allele	AF (%)	Genotype	GF (%)	Samples (n)	HWE *p* values	Fisher’s *p* value
13	SNP 1536 C > T **rs374670788**	Control (114)	C T	99 1	CC CT TT	99 1 0	113 1 0	0.962	–
		Colon (54)	C T	99 1	CC CT TT	98 2 0	53 1 0	0.945	0.540
		Bladder (16)	C T	97 3	CC CT TT	94 6 0	15 1 0	0.897	0.231
14	Mutation 1648 G > A	Control (113)	G A	100 0	GG GA AA	100 0 0	113 0 0	–	–
		Colon (64)	G A	95.3 4.7	GG GA AA	91 9 0	58 6 0	0.693	0.0019
		Lung (10)	G A	75 25	GG GA AA	50 50 0	5 5 0	0.291	0.000001
		Ovary (11)	G A	86.33 13.64	GG GA AA	73 27 0	8 3 0	0.600	0.0005
		Bladder (16)	G A	81.25 18.75	GG GA AA	63 38 0	10 6 0	0.355	0.000001
		Thyroid (9)	G A	88.89 11.11	GG GA AA	78 22 0	7 2 0	0.707	0.004
15	Mutation 1769 A > G	Control (97)	A G	100 0	AA AG GG	100 0 0	97 0 0	–	–
		Colon (37)	A G	99 1	AA AG GG	97 3 0	36 1 0	0.933	0.246
	Mutation 1804 G > A	Control (97)	A G	100 0	AA AG GG	100 0 0	97 0 0	–	–
		Colon (37)	G A	99 1	GG GA AA	97 3 0	36 1 0	0.933	0.246
	Mutation 1815 C > T	Control (97)	C T	100 0	CC CT TT	100 0 0	97 0 0		–
		Colon (37)	C T	99 1	CC CT TT	97 3 0	36 1 0	0.933	0.246
16	Mutation 1896T > A	Control (114)	T A	100 0	TT TA AA	100 0 0	114 0 0	–	–
		Colon (64)	T A	98.4 1.6	TT TA AA	90 10 0	62 2 0	0.898	0.127
		Ovary (10)	T A	95 5	TT TA AA	90 10 0	9 1 0	0.867	0.08
		Thyroid (9)	T A	77.8 22.2	TT TA AA	55.6 44.4 0	5 4 0	0.391	0.00001
	SNP 1913 C > T **rs571064657**	Control (114)	C T	99.12 0.88	CC CT TT	98.25 1.75 0	112 2 0	0.924	–

NCBI assigned new SNPs are highlited in bold

*AF* allele frequency, *GF* genotype frequency, *HWE* Hardy–Weinberg equilibrium

**Table 2:** **Analysis of polar interactions** **between EGF/EGFR in the untethered form of EGFR.** The polar interactions between EGFR wild type (3NJP) and its mutated forms R521K and V550M revealed no changes in H-bonds with mutant R521K besides 1 missing and 1 extra H-bond (highlited in bold) with the mutant V550M

EGF (ligand)-EGFR (wild type)	EGFR with (mutant R521K)	EGFR with (mutant V550M)
Lys^28^-Glu^90^	Lys^28^-Glu^90^	Lys^28^-Glu^90^
Ala^25^-Ser^99^	Ala^25^-Ser^99^	Ala^25^-Ser^99^
Asp^11^-Ser^356^	Asp^11^-Ser^356^	Asp^11^-Ser^356^
Glu^51^-Ser^468^	Glu ^51^-Ser^468^	Glu^51^-Ser^468^
Trp^50^-Lys^465^	Trp^50^-Lys^465^	Trp^50^-Lys^465^
Lys^48^-Gln^411^	Lys^48^-Gln^411^	Lys^48^-Gln^411^
*** Asp***^***46***^***-His***^***409***^ *** Lys***^***48***^***-His***^***409***^	***Asp***^***46***^***-His***^***409***^ ***Lys***^***48***^***-His***^***409***^	***Asp***^***46***^***-His***^***409***^ ***–***
Arg^41^ Arg^41^-Asp^355^	Arg^41^ Arg^41^-Asp^355^	Arg^41^ Arg^41^-Asp^355^
*** Gln***^***43***^***-Gln***^***384***^ *** Arg***^***45***^***-Gln***^***384***^	***Gln***^***43***^***-Gln***^***384***^ ***Arg***^***45***^***-Gln***^***384***^	***Gln***^***43***^***-Gln***^***384***^ ***Arg***^***45***^***-Gln***^***384***^ ***His***^***16***^***-Gln***^***384***^
Tyr^44^-His^346^	Tyr^44^-His^346^	Tyr^44^-His^346^
Cys ^33^ Asn^32^-Gly^18^	Cys^33^ Asn^32^-Gly^18^	Cys^33^ Asn^32^-Gly^18^
Cys^33^ Cys^31^-Gln^16^	Cys^33^ Cys^31^-Gln^16^	Cys^33^ Cys^31^-Gln^16^
Cys^31^Glu^40^-Thr^15^	Cys^31^Glu^40^-Thr^15^	Cys^31^Glu^40^-Thr^15^
Gly^39^-Asn^12^	Gly^39^-Asn^12^	Gly^39^-Asn^12^
Glu^40^-Ser^11^	Glu^40^-Ser^11^	Glu^40^-Ser^11^

### In silco analysis

This study focused on the interaction of EGFR variants V550M and R521K with EGF. As shown in Table 2, fifteen residues of wild type EGFR were found to interact with EGF through H-bonds. No differences in H-bonds were observed in the interface with mutant R521K. However, one H-bond was missing and one extra H-bond was identified in mutant V550M interaction with EGF (Additional file 3). Moreover, 5 residues of the EGFR were involved in internal interactions between CR1 and CR2 domains (Table 3). For mutants R521K and V550M, one H-bond was found to be missing in the interaction between CR1 and CR2 (Additional file 4). No differences in H-bonds were observed in the other alterations. SDM analysis on the human EGFR revealed that both mutants V550M and R521K were predicted to be destabilizing mutations in term of complex stability with ∆∆G values of − 1.16 and − 0.29, respectively.

**Table 3:** **Analysis of polar interactions** **between CR1/CR2 domains in the tethered form of EGFR.** The polar interaction between EGFR wild type (1NQL) and its mutated forms R521K and V550M showed 1 missing H-bond (highlited in bold)

CR1/CR2 domains (wild type)	CR1/CR2 domains (mutant R521K)	CR1/CR2 domains (mutant V550M)
Asn^247^-Glu^578^	Asn^247^-Glu^578^	Asn^247^-Glu^578^
*** Tyr***^***246***^***-Glu***^***578***^	***–***	***–***
Tyr^246^-Met^576^	Tyr^246^-Met^576^	Tyr^246^-Met^576^
Tyr^246^-Lys^585^	Tyr^246^-Lys^585^	Tyr^246^-Lys^585^
Tyr^246^-Asp^563^	Tyr^246^-Asp^563^	Tyr^246^-Asp^563^

## Discussion

While a number of studies showed that mutations of EGFR intracellular signaling region can lead to tumorigenesis, less information is available about the extracellular region of EGFR. This region is structurally instrumental to the molecule function as it contains the binding site of the antibody cetuximab successfully used in cancer therapy [26]. This study focused on the CR2 domain that was shown to interact with the CR1 domain and play a major role in the receptor’s dimerization and functions like ligand binding, growth stimulation and tyrosine kinase activation [27]. We identified 7 new gene variants in the EGFR-CR2 domain coding exons. The V550M mutation was found to be significantly associated to colon, ovary, lung, bladder and thyroid cancer samples (p < 0.05), which suggest that this mutation could be associated to tumorigenesis. The 3 novel mutations observed in exon 15 were found in the heterozygous form and only in colon cancer patients. The new alteration 1896T > A leading to a synonymous mutation (G632G) was mostly encountered in thyroid tumors but also less frequently in ovary and colon tumors. The data suggest that further study on larger sample size is mandatory to get accurate significance of the association between T/A genotype and thyroid cancer risk in Arab patients. Meanwhile, we did not observe a significant variant of the SNP rs2227983 genotypes frequencies between cancers and controls samples with over 50% being G/G in both groups. The impact of mutations on the function of protein associated to cancer can be predicted through in silico studies of its structure. Such information is crucial in understanding genotype–phenotype correlations and disease biology. The analysis of V550M substitution showed no striking differences in the interactions interface between EGF and EGFR wild type and R521K mutated form. However, SDM analysis revealed a reduction in the stabilization states of the EGFR/EGF complex for both V550M and R521K mutations. The impact of the V550M mutation on the complex stability can be described as a result of size difference, where the large methionine residue cannot fit within the available space which might disrupt the original core structure of CR2 domain and have an impact on its function. In turn this alters the interaction with its protein partner thereby affecting the signaling pathways. In the tethered (inactive) configuration of EGFR, the CR1 loop interacts closely with the CR2 domain and several side-chain to the backbone, and backbone to backbone hydrogen bonds are formed between CR1/CR2 domains. The side chain of tyrosine (Tyr^246^) is critical for both CR1/CR2 interactions, and it’s found close to the tip of the CR1 β-hairpin/loop interact via hydrogen bonds with the Glu^578^, Met^576^, Lys^585^ and Asp^563^ in the side chains of CR2 domain. Only one hydrogen bond out of five (Tyr^246^-Glu^578^) was missing in both R521K and V550M. These mutations might disrupt the intramolecular domain CR1/CR2 interactions of EGFR leading up to exposure of the EGFR dimerization arm and enhance the reaction to EGF ligand by increasing its binding affinity. Therefore, the H-bond between (Tyr^246^-Glu^578^) is crucial for the regulation of receptor activation because it’s involved in the stabilization of the receptor dimer interface, auto-inhibition, and impairment of receptor function. These data have guided our decision to raise monoclonal antibodies that distinguish the two variants R and K to ultimately develop these antibodies as an anti-cancer drug to be administered to a patient according to their rs2227983 genotype. Indeed, R521K variant is situated at the boundary of EGFR domains III and IV, at the location of the anti-cancer mAb cetuximab specific epitope [28]. This variant is known to impact the outcome of antibody-based therapy. It is also associated with the weakening of the EGFR functions as compared to the wild type [16]. The R521K variant has also been described as being associated with cancer severity in EGFR-expressing tumors, like gliomas, lung cancer and breast cancer [29–31].

## Conclusion

This study revealed new genetic variants in the EGFR-CR2 domain in cancer patient from the Arabian peninsula. The in-silico study highlighted the effect of two variants on the receptor structure–function and their eventual implication in tumorigenesis.

## Limitations

The genetic association of mutations in the EGFR extracellular domains requires validation on a larger number of patients and their family members. This will also allow haplotypes analysis. Moreover, the patients’ clinicopathological data was not accessible.

## Supplementary Information

**Additional file 1:** Primers used to amplify exons encoding CR2 sub-domain of human EGFR.

**Additional file 2:** A list of *EGFR* gene variants. The previously reported alterations found in the CR2 domain of *EGFR* gene in patients and healthy control samples from the Arabian peninsula region.

**Additional file 3:** Polar interactions between wild type and mutated EGFR with EGF (untethered monomer, 3NJP). A) Wild EGF/EGFR complex shows 15 polar interactions. B) EGF/EGFR-V550M showing 1 missing and 1 extra polar interaction. (Blue residues represent EGFR wild type; green residues represent EGF wild type).

**Additional file 4**: Polar interactions between wild type and mutated EGFR with EGF (untethered monomer, 3NJP). A) Wild CR1/CR2 shows 5 polar interactions. B) CR1/CR2-R521K showing 4 polar interactions. C) CR1/CR2-V550M showing 4 polar interactions. (Blue residues represent CR1 domain, green residues represent CR2 domain).

## Data Availability

The data sets used and/or analysed during the current study are available from the corresponding author on reasonable request.

## Abbreviations

EGFR: Epidermal growth factor receptor
FFPE: Formalin-fixed paraffin-embedded
PCR: Polymerase chain reaction
CR1: Cysteine-rich domain 1
CR2: Cysteine-rich domain 2
