# Supplementary material for: Genetic variants of the EGFR ligand-binding domain and their association with structural alterations in Arab cancer patients
Source: BMC Res Notes. 2021 Apr 19;14:146. doi: 10.1186/s13104-021-05559-y (PMC8054381; doi:10.1186/s13104-021-05559-y)
Supplement: Supplementary file 1 — Additional file 1: Primers used to amplify exons encoding CR2 sub-domain of human EGFR. [file 13104_2021_5559_MOESM1_ESM.pdf]

Table S1: Details of exons encoding CR2 sub-domain of human EGFR with primers and protocols.

Exons	Primers	Sequences	PCR product size	*Annealing temp. in all samples
**13**	EGFR-13F<br>EGFR-13R	GTCACCCAAGGTCATGGAGCACAGG<br>CAGAATGCCTGTAAAGCTATAAC	324 bp	63.8˚C
**14**	EGFR-14F<br>EGFR-14R	GTCCTGGAGTCCCAACTCCTTGAC<br>GGAAGTGGCTCTGATGGCCGTCCTG	330 bp	64.5˚C
**15**	EGFR-15F<br>EGFR-15R	AAGCAGGTGCAATCACAGAA<br>CAAACCTCGGCAATTTGTTG	421bp	63.6°C
**16**	EGFR-16F<br>EGFR-16R	CCAATCCAACATCCAGACACATAG<br>CCAGAGCCATAGAAACTTGATCAG	306 bp	62˚C

The PCR protocol for all samples include a denaturation step at 95˚C for 5 min, followed by 33 cycles of 95˚C for 1 min, *annealing temperature for 1 min, 72˚C for 1 min, a final extension step at 72˚C for 7 min. And, final hold at 10°C.
