# Supplementary material for: Genetic variants of the EGFR ligand-binding domain and their association with structural alterations in Arab cancer patients
Source: BMC Res Notes. 2021 Apr 19;14:146. doi: 10.1186/s13104-021-05559-y (PMC8054381; doi:10.1186/s13104-021-05559-y)
Supplement: Supplementary file 2 — Additional file 2: A list of EGFR gene variants. The previously reported alterations found in the CR2 domain of EGFR gene in patients and healthy control samples from the Arabian peninsula region. [file 13104_2021_5559_MOESM2_ESM.pdf]

Table S2: A list of *EGFR* gene variants. The previously reported alterations found in the CR2 domain of *EGFR* gene in cancer patients and healthy control samples from the Arabian Peninsula.

Exon	SNP ID	Nucleotide change	Protein change	Control (n=114)		Case (n=96)		*p* Value *
				Alteration	Freq. (%)	Alteration	Freq. (%)	
**13**	rs17336800	**C**1509**T**	**G**503**G**	2	1.8	3	3.1	0.419
	rs1188823834	**G**1512**C**	**Q**504**H**	0	0	3	3.1	0.093
	rs1357037344	**C**1515**A**	**V**505**V**	0	0	1	1	0.457
	rs142429250	**G**1554**A**	**P**518**P**	1	0.9	0	0	0.542
	rs2227983	**G**1562**A**	**R**521**K**	54	**47.4**	46	**47.9**	0.523
**14**	rs17290103	**T**1632**C**	**G**544**G**	1 (n=113)	0.9	1 (n=118)	0.8	0.741
**15**	rs953188147	**A**1756**G**	**I**586**V**	1 (n=97)	1	0 (n=37)	0	0.723
	rs17290162	**G**1788**A**	**P**596**P**	1 (n=97)	1	0 (n=37)		0.723
	rs17290169	**C**1839**T**	**A**613**A**	22 (n=97)	22.7	1 (n=37)	2.7	**0.003**
**16**	rs2227984	**T**1887**A**	**T**629**T**	82	71.9	81 (n=120)	67.5	0.276

Freq: frequency, *p*-value: Fisher's exact test.
