# Supplementary material for: Genetic variants of the EGFR ligand-binding domain and their association with structural alterations in Arab cancer patients
Source: BMC Res Notes. 2021 Apr 19;14:146. doi: 10.1186/s13104-021-05559-y (PMC8054381; doi:10.1186/s13104-021-05559-y)

Fig. S1: Polar interactions between wild type and mutated EGFR with EGF (untethered monomer, 3NJP). A) Wild EGF/EGFR complex shows 15 polar interactions. B) EGF/EGFR-V550M showing 1 missing and 1 extra polar interaction. (Blue residues represent EGFR wild type; green residues represent EGF wild type).

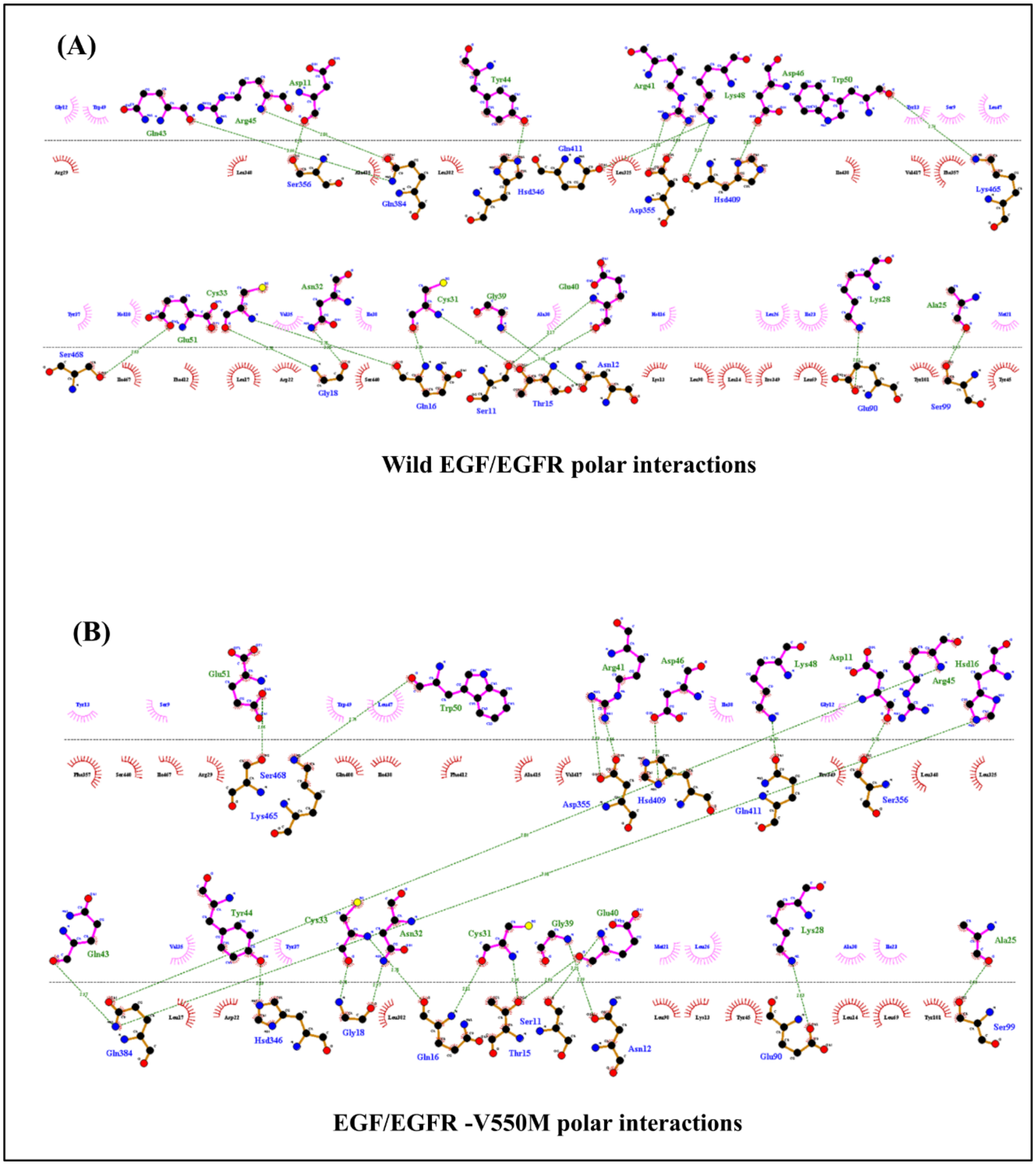

Supplement: Supplementary file 3 — Additional file 3: Polar interactions between wild type and mutated EGFR with EGF (untethered monomer, 3NJP). A) Wild EGF/EGFR complex shows 15 polar interactions. B) EGF/EGFR-V550M showing 1 missing and 1 extra polar interaction. (Blue residues represent EGFR wild type; green residues represent EGF wild type). [file 13104_2021_5559_MOESM3_ESM.pdf]
