# Supplementary material for: Genetic variants of the EGFR ligand-binding domain and their association with structural alterations in Arab cancer patients
Source: BMC Res Notes. 2021 Apr 19;14:146. doi: 10.1186/s13104-021-05559-y (PMC8054381; doi:10.1186/s13104-021-05559-y)

Fig. S2: Polar interactions between wild type and mutated EGFR with EGF (untethered monomer, 3NJP). A) Wild CR1/CR2 shows 5 polar interactions. B) CR1/CR2-R521K showing 4 polar interactions. C) CR1/CR2-V550M showing 4 polar interactions. (Blue residues represent CR1 domain, green residues represent CR2 domain).

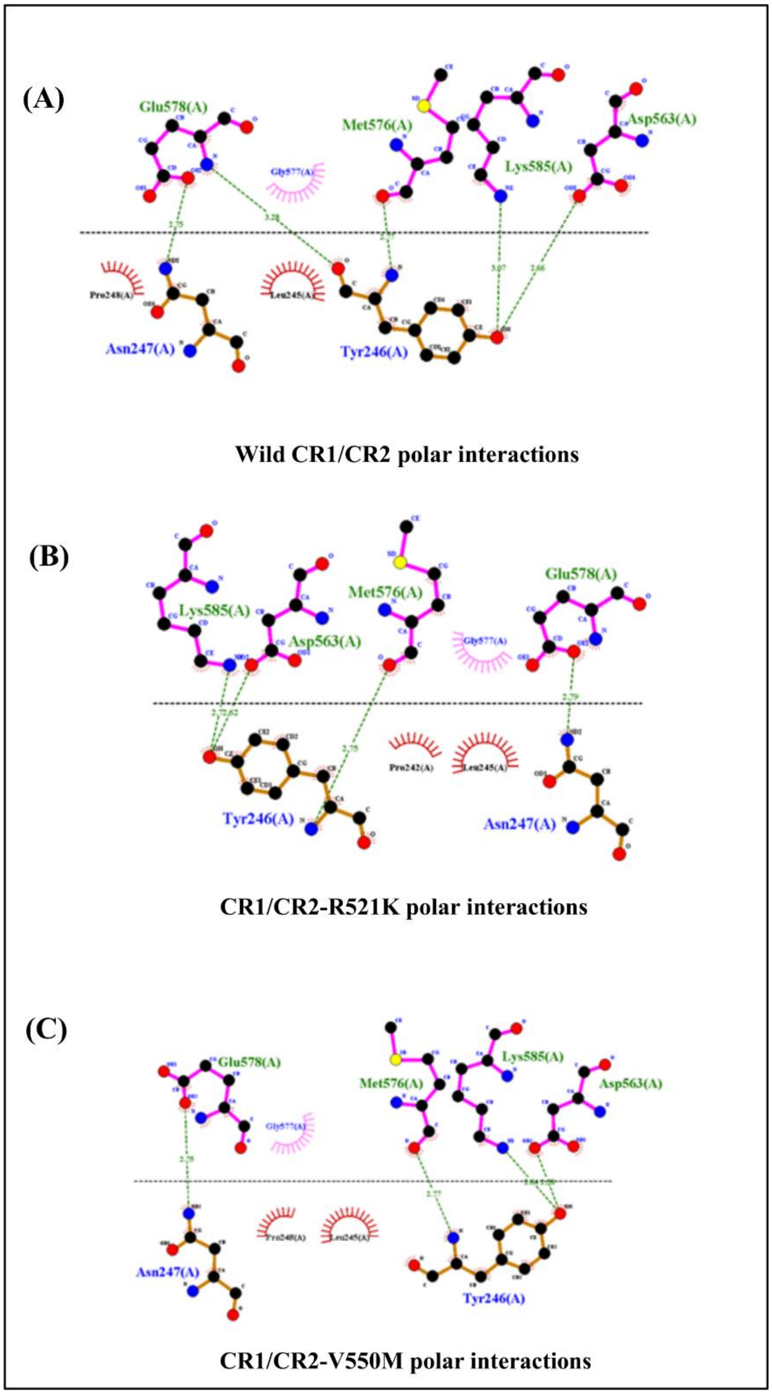

Supplement: Supplementary file 4 — Additional file 4: Polar interactions between wild type and mutated EGFR with EGF (untethered monomer, 3NJP). A) Wild CR1/CR2 shows 5 polar interactions. B) CR1/CR2-R521K showing 4 polar interactions. C) CR1/CR2-V550M showing 4 polar interactions. (Blue residues represent CR1 domain, green residues represent CR2 domain). [file 13104_2021_5559_MOESM4_ESM.pdf]
